# A Unique Case of Cryptococcus and Histoplasmosis Co-infection in an HIV-negative Male on Chronic Steroid Therapy

**DOI:** 10.7759/cureus.4654

**Published:** 2019-05-14

**Authors:** Samia Asif, Joseph Bennett, Rebecca R Pauly

**Affiliations:** 1 Internal Medicine, University of Missouri Kansas City (UMKC), Kansas City, USA; 2 Internal Medicine, Virginia Tech Carilion School of Medicine, Roanoke, USA

**Keywords:** cryptococcus neoformans, histoplasma, chronic steroid therapy

## Abstract

Histoplasmosis and cryptococcosis are systemic fungal diseases frequently encountered in immunocompromised hosts, particularly in patients with HIV/AIDS with low CD4 counts. However, co-infection with histoplasmosis and cryptococcosis is an uncommon clinical scenario, hence carrying the risk of under diagnosis by medical professionals. For instance, when one infection is identified, most health professionals will have a low suspicion for an additional co-infection. Here, we report the case of a 71-year-old gentleman with a new diagnosis of myasthenia gravis (MG) requiring recent steroid therapy who presented with recurrent respiratory symptoms despite treatment for community acquired pneumonia. Bronchoscopy and bronchoalveolar lavage (BAL) were performed; BAL samples revealed presence of *Cryptococcus neoformans* and histoplasma antigen (Ag). Serum cryptococcal Ag and urine histoplasma Ag returned positive as well. The patient then required inpatient treatment with amphotericin B, with eventual transition to oral fluconazole at discharge. Pulmonology and Infectious disease consults assisted in appropriate diagnosis and management of this rare presentation. Given the high prevalence of immunocompromised states in a myriad of medical co-morbidities, it is important to highlight this case to create awareness regarding possibility of concomitant systemic fungal diseases.

## Introduction

Cryptococcosis and histoplasmosis are fungal infections most commonly seen in patients with AIDS [1]. Concurrent infection with histoplasmosis and cryptococcosis, however, is a rare entity even in these group of immunocompromised individuals [1]. The rarity of the co-infection leads to the unfortunate possibility that the diagnosis of one fungal infection may lead to low suspicion for the presence of a second infection. This in turn will lead to inadequate treatment and hence poorer patient outcomes. The fact that patients having cryptococcosis-histoplasmosis co-infections are mostly immunocompromised individuals, an overlooked diagnosis will worsen clinical outcomes. Here we present a case of an elderly, HIV-negative gentleman who was diagnosed with a synchronous pulmonary cryptococcosis and histoplasmosis.

## Case presentation

A 71-year-old gentleman with a past medical history of type 2 diabetes mellitus had been diagnosed with seronegative myasthenia gravis (MG). He was subsequently started on prednisone. He eventually received intravenous immunoglobulins (IVIG) every four weeks. He had undergone a CT chest angiogram for worsening dyspnea three months prior that had shown bilateral, acute, large pulmonary emboli. IVIG was felt to have contributed to this thrombotic event. He was discharged on apixaban. The patient was now evaluated in the ER for a one-day history of chest pain with dyspnea, 18 months after initial diagnosis of MG. He was afebrile with blood pressure of 108/67 mmHg, heart rate 70 beats per minute (bpm), and with oxygen saturation of 95% on 2 liters/minute (L/min) oxygen via nasal cannula. Physical examination was remarkable for bibasilar crackles. He was noted to have leukocytosis with a white cell count of 11,300 mm^3^ including a 95% neutrophilic predominance. His creatinine was 1.0 mg/dL.

**Figure 1:**
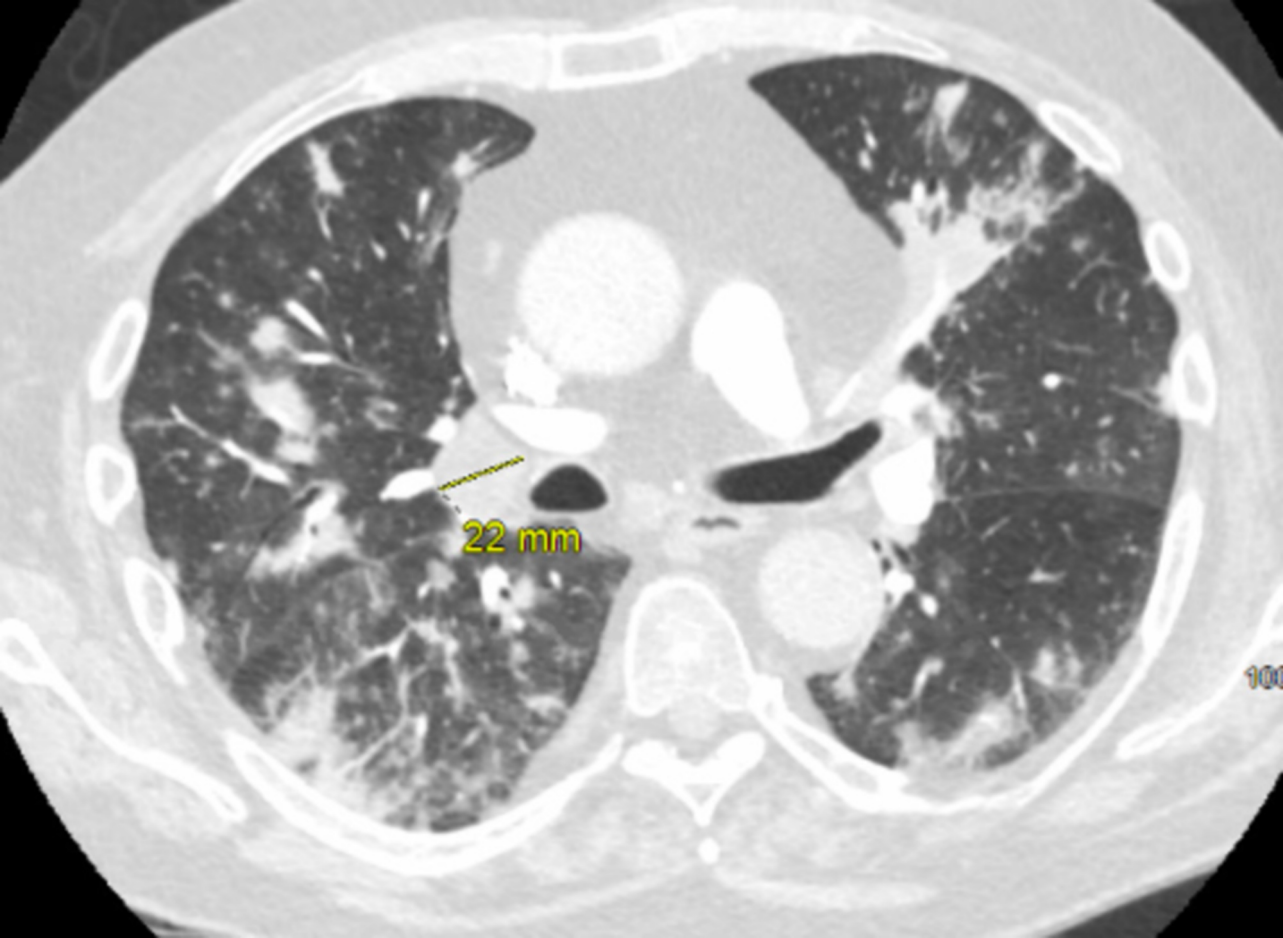
CT angiogram chest showing multifocal infiltrates and prominent right hilar lymphadenopathy.

A repeat CT chest angiogram showed multifocal infiltrates in all five lung lobes with subtle ground glass opacity surrounding most infiltrates. The patient tested negative for HIV. He was started on broad-spectrum antibiotics including vancomycin, piperacillin-tazobactam, and ciprofloxacin. At the time, he reported taking prednisone 20 mg daily and mycophenolate 1000 mg twice daily for MG, in addition to pyridostigmine. Bronchoscopy was performed to rule out atypical infections such as pneumocystis jiroveci pneumonia (PJP). Urinary streptococcal, histoplasmosis, and legionella antigens (Ag) were ordered. In addition, viral respiratory polymerase chain reaction (PCR) testing, bacterial cultures, fungal cultures, and acid-fast bacilli (AFB) cultures were performed on the bronchoalveolar lavage (BAL). Antibiotics were subsequently de-escalated to levofloxacin as bacterial respiratory and BAL cultures returned negative. He was discharged home. However, he was re-admitted within a week with acute hypoxemic respiratory failure. A repeat CT chest angiogram now showed diffuse nodular infiltrates that were worsening despite having recently received antimicrobial therapy (Figure 1). 

He also reported two episodes of hemoptysis a few days prior to this presentation. No antibacterial agents were started at this point. He was afebrile. Given concerns for possible vasculitis, screening anti-neutrophil cytoplasmic antibodies (ANCA) and autoimmune panel were ordered. Meanwhile, results from his prior BAL were reviewed and returned positive for histoplasma antigen (Ag). Silver stain was positive for budding yeast, which was subsequently identified as Cryptococcus. Urine histoplasma Ag then returned positive as well. Infectious disease (ID) team was consulted. Management was started for disseminated histoplasmosis with pulmonary manifestation. Given he had MG and was on immunosuppressive agents, he was considered high-risk for opportunistic infections.

The patient was started on IV liposomal amphotericin B with close monitoring of renal function, magnesium, and potassium. Serum cryptococcal antigen was ordered which resulted positive as well. Amphotericin was continued for one week until it had to be discontinued secondary to acute kidney injury, with creatinine increased to 2.1 mg/dL from his baseline of 1.0 mg/dL. He was then transitioned to fluconazole. Lumbar puncture was performed to evaluate for further dissemination of disease. However, cerebrospinal fluid (CSF) analysis was negative for cryptococcal Ag and CSF cultures were negative subsequently. Hence, the patient was discharged on fluconazole with a plan to follow-up as outpatient in ID clinic.

## Discussion

Our case reports synchronous pulmonary histoplasmosis and cryptococcosis in an elderly gentleman with type 2 diabetes who received long-term corticosteroid therapy for MG. Literature review with search terms histoplasmosis, cryptococcosis, co-infection and simultaneous; starting from 1940 to date, on PubMed and Medline, to best of our knowledge, revealed only 11 prior reported cases of cryptococcosis-histoplasmosis co-infection. Of these, all but three were associated with HIV with low CD4 counts [1]. All but two cases were reported from North America and Latin America; the others included one from India and one from France. Of the three cases not associated with HIV, one patient was diabetic; second was diabetic with autoimmune thrombocytopenia and received steroids and in the third case, no risk factors were identified. The patient in our case was elderly, diabetic and had received high dose steroids for a myasthenia crisis, all potential risk factors.

For both Cryptococcus and histoplasma, mode of acquisition of infection is via inhalation. An intact immune system usually controls the infection [2-3]. For Cryptococcus, about 40% infected individuals develop pulmonary symptoms, mostly similar to those observed with bacterial pneumonia; in less than 1% cases, there is extra thoracic dissemination to meninges, bones, joints, skin, or soft tissues [4]. In contrast, histoplasmosis is usually asymptomatic if a patient is immunocompetent; however, in cases of immunodeficiency, 95% patients develop symptomatic infection [5]. Treatment involves parenteral amphotericin B until patient improves, followed by itraconazole 200 mg daily, with duration of treatment depending on clinical presentation, severity of illness, and patient response to treatment. Fluconazole is a second-line agent for treatment of histoplasmosis, but higher doses of 400-800 mg daily are typically needed [6-7].

For Cryptococcus, serum cryptococcal Ag should be performed, particularly in immunocompromised patients [8]. Meningoencephalitis is the most common manifestation of disseminated Cryptococcus neoformans. For diagnosis of cryptococcal meningitis, CSF analysis including: India ink staining, cryptococcal PCR, CSF culture, and Cryptococcal antigen testing should be performed [9-10]. Treatment involves induction with amphotericin B in combination with flucytosine or fluconazole in cases of CNS involvement, followed by consolidation and maintenance with fluconazole or itraconazole; usually up to one-year duration of treatment. For non-CNS infections, treatment is outlined mainly for pulmonary infections and involves treatment with azoles, such as fluconazole or itraconazole [11-12].

Identification of each of the due causative organisms is essential because this has significant implications for optimal management. For histoplasmosis, drug of choice is itraconazole; if for any reason, fluconazole is opted for instead, dose required is much higher (400-800 mg daily) than that recommended to be used for cryptococcosis. More importantly, if serum cryptococcal Ag is positive, then CSF analysis has to be done to rule out cryptococcal meningoencephalitis, because while itraconazole can be used for pulmonary cryptococcal infection, itraconazole does not have adequate CSF penetration and would not be used if that CSF analysis was positive for cryptococcal Ag.

It is essential to highlight this case given that all medicine practitioners frequently manage patients in immune compromised states: such as those on chronic steroid therapy for a myriad of disease states such as rheumatoid arthritis, elderly, solid organ transplant recipients or patients with hematological malignancies and not just those with HIV with low CD4 counts.

## Conclusions

Co-infections are rare; hence diagnosis of one fungal disease may result in inadequate evaluation and failure to diagnose the other organism. Management in such complicated situations may also require specialty referrals. Hence, it is important to report these cases for the knowledge of all general practitioners. 
